# Quantum State Reduction by Matter-Phase-Related Measurements in Optical Lattices

**DOI:** 10.1038/srep42597

**Published:** 2017-02-22

**Authors:** Wojciech Kozlowski, Santiago F. Caballero-Benitez, Igor B. Mekhov

**Affiliations:** 1 Department of Physics, Clarendon Laboratory, University of Oxford, Parks Road, Oxford OX1 3PU, United Kingdom; 2 CONACYT, Instituto Nacional de Astrofísica, Óptica y Electrónica, Calle Luis Enrique Erro No. 1, Sta. María Tonantzintla, Pue. CP 72840, México; 3 St. Petersburg State University, Universitetsky pr. 26, 198504 St. Petersburg, Russia

## Abstract

A many-body atomic system coupled to quantized light is subject to weak measurement. Instead of coupling light to the on-site density, we consider the quantum backaction due to the measurement of matter-phase-related variables such as global phase coherence. We show how this unconventional approach opens up new opportunities to affect system evolution. We demonstrate how this can lead to a new class of final states different from those possible with dissipative state preparation or conventional projective measurements. These states are characterised by a combination of Hamiltonian and measurement properties thus extending the measurement postulate for the case of strong competition with the system’s own evolution.

Ultracold gases trapped in optical lattices is a very successful and interdisciplinary field of research^1^,^2^. Whilst normally the atoms are manipulated using classical light beams there is a growing body of work based on coupling such systems to quantised optical fields exploring the ultimate quantum level of light-matter coupling^3^,^4^. This new regime of interactions has already led to a host of fascinating phenomena, such as novel methods of non-destructive probing of quantum states^5^,^6^,^7^,^8^,^9^,^10^,^11^,^12^,^13^,^14^, new quantum phases and light-matter entanglement^15^,^16^,^17^,^18^,^19^,^20^,^21^,^22^,^23^, or an entirely new class of many-body dynamics due to measurement backaction^24^,^25^,^26^,^27^,^28^,^29^,^30^,^31^. Furthermore, recent experimental breakthroughs in coupling an optical lattice to a cavity demonstrate the significant interest in studying this ultimate quantum regime of light-matter interaction^32^,^33^.

Light scatters due to its interaction with the dipole moment of the atoms which for off-resonant light results in an effective coupling with atomic density, not the matter-wave amplitude. Therefore, it is challenging to couple light to the phase of the matter-field, as is typical in quantum optics for optical fields. Most of the existing work on measurement couples directly to atomic density operators^3^,^11^,^26^,^27^,^34^. However, it has been shown that it is possible to couple to the the relative phase differences between sites in an optical lattice by illuminating the bonds between them^13^,^20^,^21^,^22^,^23^,^35^. This is a multi-site generalisation of previous double-well schemes^36^,^37^,^38^,^39^,^40^, although the physical mechanism is fundametally different as it involves direct coupling to the interference terms caused by atoms tunnelling rather than combining light scattered from different sources.

Coupling to phase observables in lattices has been proposed and considered in the context of nondestructive probing and quantum optical potentials. In this paper, we go beyond any previous work by studying this new feature of optical lattice cavity systems in the context of measurement backaction. The quantum trajectory approach to backaction induced dynamics is not new in general and has attracted significant experimental interest in single atom cavity^41^ and single qubit circuit^42^,^43^ QED systems. However, its study in the context of many-body dynamics is much more recent and has attracted significant theoretical interest over the past years^3^,^7^,^24^,^29^,^44^,^45^,^46^,^47^,^48^. Here, it is the novel combination of measurement backaction as the physical mechanism driving the dynamics and phase coherence as the observable, which the optical fields couple to, that provides a completely new opportunity to affect and manipulate the quantum state.

In this paper we begin by presenting a simple quantum gas example. In the second part we generalize our model and show a novel type of a projection due to measurement which occurs even when there is significant competition with the Hamiltonian dynamics. This projection is fundamentally different to dissipative steady states, standard formalism eigenspace projections or the quantum Zeno effect^49^,^50^,^51^,^52^,^53^ thus providing an extension of the measurement postulate to dynamical systems subject to weak measurement. Such a measurement-based preparation is unobtainable using the dissipative state engineering, as the dissipation would completely destroy the coherence in this case.

## Results

### Quantum gas model

We consider measurement of an ultracold gas of *N* bosons trapped in an optical lattice with period *a* and *M* sites^3^. We focus on the one-dimensional case, but the general concept can be easily applied to higher dimensions. The isolated system is described by the Bose-Hubbard model with the Hamiltonian


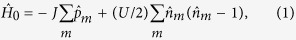


where 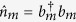 is the number operator at site *m, b*_*m*_ annihilates an atom at site *m*, 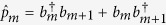, *J* is the atom hopping amplitude and *U* the on-site interaction.

The atoms are illuminated with an off-resonant beam and light scattered at a particular angle is selected and enhanced by a cavity with decay rate *κ*^54^,^55^,^56^. Just like in classical optics for light amplitude, the Heisenberg annihilation operator of the scattered light is given by 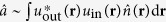, where 
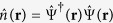
 is the atomic density operator, 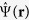 is the operator that annihilates a boson at ***r***, and *u*_in,out_(**r**) are the light mode functions for the incoming and scattered beams. Expanding the matter-field operator in terms of the Wannier functions of the lowest band, 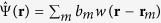, we can write 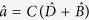
3,7, where *C* is the Rayleigh scattering coefficient and


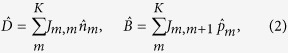


the sum is over *K* illuminated sites, and


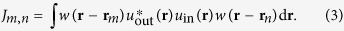


We will consider the case when the quantum potential due to the cavity light field is negligible (cavity detuning must be small compared to *κ*^20^), but the photons leak from the cavity and thus affect the system via measurement backaction instead^3^,^29^. This process can be modelled using a quantum trajectory approach where each experimental run is simulated using a stochastic Schrödinger equation. Following the formalism presented in ref. 29 the system can be shown to evolve according to 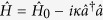 and the jump operator 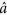 is applied to the wave function whenever a photon is detected. In a trajectory simulation the photodetection times are determined using a Monte-Carlo method. Measurement backaction affects the optical field which is entangled with the atoms and thus the quantum gas is also affected, just like the particles in the Einstein-Podolsky-Rosen thought experiment are affected by measurements on its pair^57^.

In general, it is easier for the light to couple to atom density that is localised within the lattice rather than the density within the bonds, i.e. in between the lattice sites. This means that in most cases 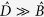 and thus 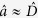. However, it is possible to arrange the light geometry in such a way that scattering from the atomic density operators within a lattice site is suppressed leading to a situation where light is only scattered from these bonds leading to an effective coupling to phase-related observables, 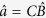
13. This does not mean that light actually scatters from the matter phase. Light scatters due to its interaction with the dipole moment of the atoms which for off-resonant light and thus the scattering is always proportional to the density distribution. However, in an optical lattice, the interference of matter waves between neighbouring sites leads to density modulations which allows us to indirectly measure these phase observables. A brief summary based on ref. 13 on how this is achieved is available in the Supplementary Information. Here, we will summarise the results and focus on the effects of measurement backaction due to such coupling.

**Figure 1:**
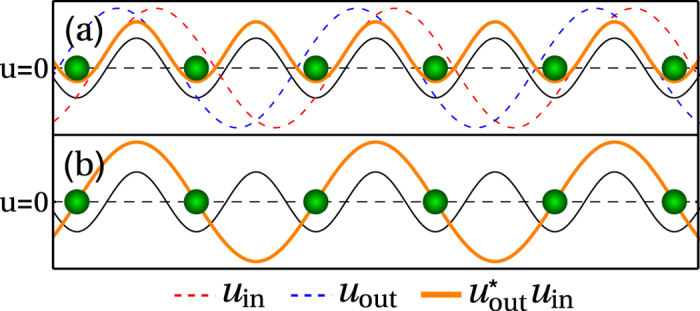
Light field arrangements which maximise coupling, 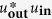, between lattice sites. The thin black line indicates the trapping potential (not to scale). (**a**) Arrangement for the uniform pattern *J*_*m,m*+1_ = *J*_1_. (**b**) Arrangement for spatially varying pattern *J*_*m,m*+1_ = (−1)^*m*^*J*_2_; here *u*_in_ = 1 so it is not shown and *u*_out_ is real thus 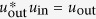.

If we consider both incoming and outgoing beams to be standing waves, 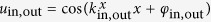 we can suppress the 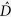-operator contribution by crossing the beams at angles such that *x*-components of the wavevectors are 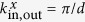, and the phase shifts satisfy *φ*_in_ + *φ*_out_ = *π* and 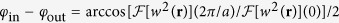, where 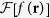 denotes a Fourier transform of *f(**r***)^13^. For clarity, this arrangement is illustrated in Fig. 1(a). This ensures that *J*_*m,m*_ = 0 whilst


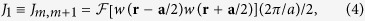


a constant, and thus 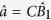 (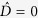, 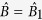) with


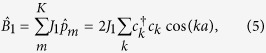


where the second equality follows from converting to momentum space via 
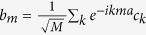
 and *c*_*k*_ annihilates an atom with momentum *k*.

In order to correctly describe the dynamics of a single quantum trajectory we have introduced a non-Hermitian term to the Hamiltonian, 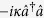. As the jump operator itself, 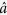 is linearly proportional to the atom density, the new term introduces a quadratic atom density term on top of the nonlocality due to the global nature of the probing. Therefore, in order to focus on the competition between tunnelling and measurement backaction we do not consider the other (standard) nonlinearity due to the atomic interactions: *U* = 0. Therefore, 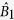 is proportional to the Hamiltonian and both operators have the same eigenstates, i.e. Fock states in the momentum basis. We can thus rewrite as


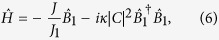


which will naturally be diagonal in the 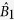 basis. Since it’s already diagonal we can easily solve its dynamics and show that the probability distribution of finding the system in an eigenspace with eigenvalue *B*_1_ after *n* photocounts at time *t* is given by


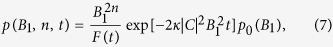


where *p*_0_(*B*_1_) denotes the initial probability of observing *B*_1_^7^,^44^,^45^ and *F(t*) is the normalisation factor. This distribution has peaks at 
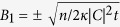
 and an initially broad distribution will narrow down around these two peaks with time and successive photocounts. The final state is in a superposition, because we measure the photon number, 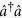 and not field amplitude. Therefore, the measurement is insensitive to the phase of 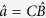 and we get a superposition of ±*B*_1_. This means that the matter is still entangled with the light as the two states scatter light with different phase which the photocount detector cannot distinguish. However, this is easily mitigated at the end of the experiment by switching off the probe beam and allowing the cavity to empty out or by measuring the light phase (quadrature) to isolate one of the components^3^,^7^,^14^. Interestingly, this measurement will establish phase coherence across the lattice, 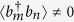, in contrast to density based measurements where the opposite is true, Fock states with no coherences are favoured.

Unusually, we do not have to worry about the timing of the quantum jumps, because the measurement operator commutes with the Hamiltonian. This highlights an important feature of this measurement - it does not compete with atomic tunnelling, and represents a quantum nondemolition (QND) measurement of the phase-related observable^58^. This is in contrast to conventional density based measurements which squeeze the atom number in the illuminated region and thus are in direct competition with the atom dynamics (which spreads the atoms), thus requiring strong couplings for a projection^29^. Here a projection is achieved at any measurement strength which allows for a weaker probe and thus effectively less heating and a longer experimental lifetime.

It is also possible to achieve a more complex spatial pattern of *J*_*m,m*+1_ 13. This way the observable will no longer commute with the Hamiltonian (and thus will no longer be QND), but will still couple to the phase related operators. This can be done by tuning the angles such that the wavevectors are 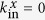 and 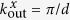 and the phase shift of the outgoing beam is *φ*_out_ = ±*π/d*. This yields


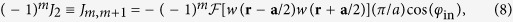


where *J*_2_ is a constant. Now 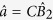 (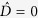, 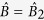) and the resulting coupling pattern is shown in Fig. 1(b). The operator 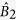 is given by,


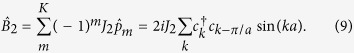


Note how the measurement operator now couples the momentum mode *k* with the mode *k* − *π/a*.

The measurement operator no longer commutes with the Hamiltonian so we do not expect there to be a steady state as before. In order to understand the measurement it will be easier to work in a basis in which it is diagonal. We perform the transformation 
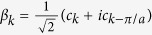
, 
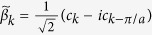
, which yields the following forms of the measurement operator and the Hamiltonian:


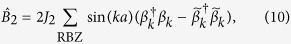



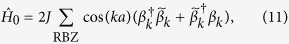


where the summations are performed over the reduced Brilluoin Zone (RBZ), 0 < *k* ≤ *π/a*, to ensure the transformation is canonical. We see that the measurement operator now consists of two types of modes, *β*_*k*_ and 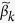, which are superpositions of two momentum states, *k* and *k* − *π/a*. Note how a spatial pattern with a period of two sites leads to a basis with two modes whilst a uniform pattern had only one mode, *c*_*k*_.

**Figure 2:**
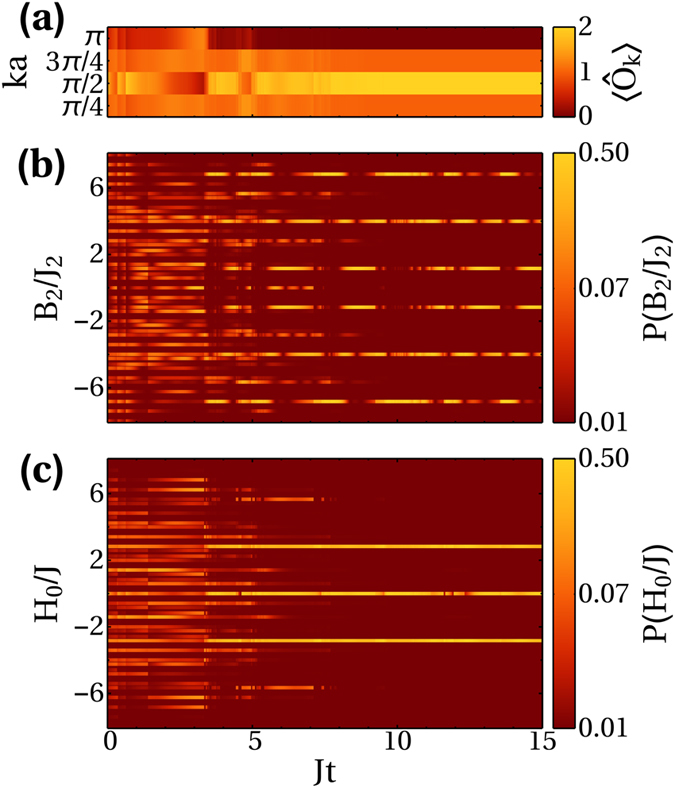
Subspace projections. Projection to a 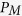 space for four atoms on eight sites with periodic boundary conditions. The parameters used are *J* = 1, *U* = 0, *κ*|*C*|^2^ = 0.1, and the initial state was |0, 0, 1, 1, 1, 1, 0, 0〉. (**a**) The 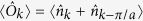 distribution becomes fully confined to its subspace at *Jt* ≈ 8 indicating the system has been projected. (**b**) Populations of the 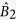 eigenspaces. (**c**) Population of the 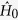 eigenspaces. Once the projection is achieved at *Jt* ≈ 8 we can see from (**b,c**) that the system is not in an eigenspace of either 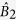 or 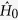, but it becomes confined to some subspace. The system has been projected onto a subspace, but it is neither that of the measurement operator or the Hamiltonian.

Trajectory simulations confirm that there is no steady state. However, unexpectedly, for each trajectory we observe that the dynamics always ends up confined to some subspace as seen in Fig. 2 which is not the same for each trajectory. In general, this subspace is not an eigenspace of the measurement operator or the Hamiltonian. In Fig. 2(b) it in fact clearly consists of multiple measurement eigenspaces. This clearly distinguishes it from the typical projection formalism. It is also not the quantum Zeno effect which predicts that strong measurement can confine the evolution of a system as this subspace must be an eigenspace of the measurement operator^49^,^50^,^51^,^52^,^53^. Furthermore, the projection we see in Fig. 2 occurs for even weak measurement strengths compared to the Hamiltonian’s own evolution, a regime in which the quantum Zeno effect does not happen. It is also possible to dissipatively prepare quantum states in an eigenstate of a Hamiltonian provided it is also a dark state of the jump operator, 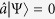 59. However, this is also clearly not the case here as the final state in Fig. 2(c) is not only not confined to a single measurement operator eigenspace, it also spans multiple Hamiltonian eigenspaces. Therefore, the dynamics induced by 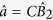 projects the system into some subspace, but since this does not happen via any of the mechanisms described above it is not immediately obvious what this subspace is.

A crucial point is that whilst single quantum trajectories might not have a steady state, for dissipative systems the density matrix will in general have a steady state which can undergo phase transitions as the dissipative parameters are varied^60^. If we were to average over many trajectories we would obtain such a steady state for this system. However, we are concerned with measurement and not dissipation. Whilst both are open systems, having knowledge of the measurement outcome from the photodetector means we deal with pure states that are the outcomes of individual measurements rather than an ensemble average over all possible outcomes. This can reveal physical effects which would be lost in a mixed state. The example in Fig. 2 shows how a single quantum trajectory can become confined yet never approach any steady state - measurement and tunnelling still compete, albeit in a limited subspace. This subspace will not in general be the same for each experimental trajectory, but once the subspace is chosen, the system will remain there. This is analogous to a QND measurement in which a system after the first projection will remain in its chosen eigenstate, but this eigenstate is not determined until the first projection takes place. However, if we were to look at the dissipative steady state (by averaging expectation values over many quantum trajectories), we would not see these subspaces at all, because the mixed state is an average over all possible outcomes, and thus an average over all possible subspaces which on a single trajectory level are mutually exclusive. Therefore, here we will consider only individual experimental runs, which are not steady states themselves, but rather the individual pure state components of the dissipative steady state that are obtained via the weak measurement of 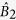.

### General model for the projection

To understand this dynamics we will look at the master equation for open systems described by the density matrix, 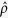,


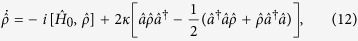


where 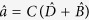 as before. This equation describes the state of the system if we discard all knowledge of the outcome which is effectively an average over all possible stochastic quantum trajectories. The commutator describes coherent dynamics due to the isolated Hamiltonian and the remaining terms are due to measurement. This is a convenient way to find features of the dynamics common to every measurement trajectory.

We define the projectors of the measurement eigenspaces, *P*_*m*_, which have no effect on any of the (possibly degenerate) eigenstates of 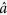 with eigenvalue *a*_*m*_, but annihilate everything else, thus 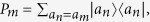 where |*a*_*n*_〉 is an eigenstate of 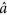 with eigenvalue *a*_*n*_. Note that since 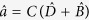 these projectors act on the matter state. This allows us to decompose the master equation in terms of the measurement basis as a series of equations 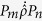. For *m* = *n*, 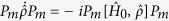, the measurement terms disappear which shows that a state in a single eigenspace is unaffected by observation. On the other hand, for *m* ≠ *n* the Hamiltonian evolution actively competes against measurement. In general, if 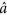 does not commute with the Hamiltonian then a projection to a single eigenspace *P*_*m*_ is impossible.

We now define a new type of projector 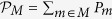, such that 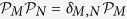 and 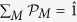 where *M* denotes some arbitrary subspace. The first equation implies that the subspaces can be built from *P*_*m*_ whilst the second and third equation specify that these projectors do not overlap and that they cover the whole Hilbert space. Furthermore, we will also require that 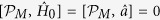. The second commutator simply follows from the definition of 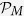, but the first one is non-trivial. However, if we can show that 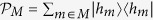, where |*h*_*m*_〉 is an eigenstate of 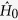 then the commutator is guaranteed to be zero. Note that we always have the trivial case where all these conditions are satisfied and that is when there is only one such projector 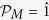.

Assuming that it is possible to have non-trivial cases where 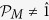 we can write the master equation as


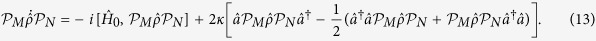


Crucially, thanks to the commutation relations we were able to divide the density matrix in such a way that each submatrix’s time evolution depends only on itself. When we partitioned the matrix with *P*_*m*_ the fact that the projectors did not commute with the operators meant that we had terms of the form 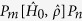 which couple many different 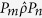 submatrices with each other.

We note that when *M* = *N* the equations for 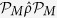 will include subspaces unaffected by measurement, i.e. 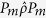. Therefore, parts of the 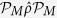 submatrices will also remain unaffected by measurement. However, the submatrices 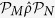, for which *M* ≠ *N*, are guaranteed to not contain measurement-free subspaces thanks to the orthogonality of 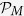. Therefore, for *M* ≠ *N* all elements of 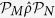 will experience a non-zero measurement term whose effect is always dissipative/lossy. Furthermore, these coherence submatrices 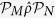 are not coupled to any other part of the density matrix and so they can never increase in magnitude; the remaining coherent evolution is unable to counteract the dissipative term without an ‘external pump’ from other parts of the density matrix. The combined effect is such that all 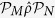 for which *M* ≠ *N* will always go to zero.

When all these cross-terms vanish, we are left with a density matrix that is a mixed state of the form 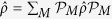. Since there are no coherences, 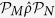, this state contains only classical uncertainty about which subspace, 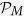, is occupied - there are no quantum superpositions between different 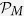 spaces. Therefore, in a single measurement run we are guaranteed to have a state that lies entirely within a subspace defined by 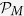.

Before moving on to a specific example we will briefly discuss the regime of validity of this result. In principle, this should be applicable to any open system that can be described by the master equation in Eq. (12) as the projectors *P*_*m*_ can be constructed for any jump operator. The peculiar form of our operators, namely that 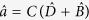, simply allows us to limit our system to just the matter state, but is in general not necessary to obtain the result above. In fact, QND measurements, such as the one seen in the previous section, are another special case where each of the new projectors 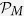 is made of a sum of projectors *P*_*m*_ in a single degenerate subspace. Therefore, the existence of these emergent subspaces relies on exactly the same physical approximations as the master equation and is simply one of the properties of Markovian open systems. However, the existence of these trivial cases alone does not justify the introduction of a new set of projectors. Furthermore, the derivation alone does not help us in identifying what systems might have non-trivial subspaces or whether any even exist. Since this result applies to any system described by a master equation which will always exhibit the trivial cases of the identity and QND measurement projectors, it is unclear whether it is in general possible to predict which Hamiltonians might have these non-trivial emergent subspaces.

However, it turns out that such a non-trivial case is indeed possible for our 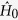 and 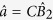 and we can see the effect in Fig. 2. Whilst the result is general and applicable to any Markovian system, we identified the first non-trivial case only for phase observable measurements in an optical lattice. This is thanks to the fact that the measurement operator is similar in form to the Hamiltonian, but at the same time it does not commute with it (otherwise we would have a QND measurement).

In Fig. 2 we can clearly see how a state that was initially a superposition of a large number of eigenstates of both operators becomes confined to some small subspace that is neither an eigenspace of 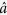 or 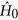. In this case the projective spaces, 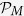, are defined by the parities (odd or even) of the combined number of atoms in the *β*_*k*_ and 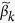 modes for different momenta 0 < *k* < *π/a* that are distinguishable to 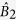. The explanation requires careful consideration of where the eigenstates of the two operators overlap and is described in Section S3 of the Supplementary Information.

**Figure 3:**
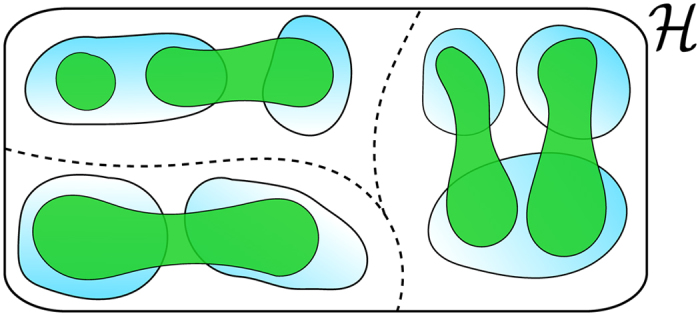
A visual representation of the projection spaces of the measurement. The light blue areas (bottom layer) are *R*_*m*_, the eigenspaces of 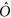. The green areas are measurement eigenspaces, *P*_*m*_, and they overlap non-trivially with the *R*_*m*_ subspaces. The 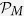 projection space boundary (dashed line) runs through the Hilbert space, 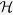, where there is no overlap between *P*_*m*_ and *R*_*m*_.

To understand the physical meaning of these projections we define an operator 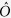 with eigenspace projectors *R*_*m*_, which commutes with both 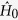 and 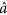. Physically this means that 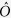 is a compatible observable with 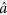 and corresponds to a quantity conserved by the Hamiltonian. The fact that 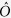 commutes with the Hamiltonian implies that the projectors can be written as a sum of Hamiltonian eigenstates 
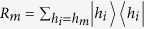
 and thus a projector 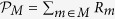 is guaranteed to commute with the Hamiltonian and similarly since 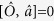 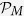 will also commute with 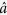 as required. Therefore, 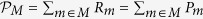 will satisfy all the necessary prerequisites. This is illustrated in Fig. 3.

In the simplest case the projectors 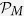 can consist of only single eigenspaces of 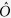, 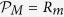. The interpretation is straightforward - measurement projects the system onto a eigenspace of an observable 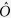 which is a compatible observable with 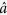 and corresponds to a quantity conserved by the coherent Hamiltonian evolution. However, this may not be possible and we have the more general case when 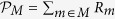. In this case, one can simply think of all *R*_*m*∈*M*_ as degenerate just like eigenstates of the measurement operator, 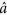, that are degenerate, can form a single eigenspace *P*_*m*_. However, these subspaces will correspond to different eigenvalues of 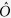 distinguishing it from conventional projections.

In our case, it is apparent from the form of 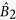 and 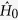 that 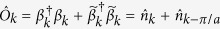 commutes with both operators for all *k*. Thus, we can easily construct 
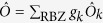
 for any arbitrary *g*_*k*_. Its eigenspaces, *R*_*m*_, can then be easily constructed and their relationship with *P*_*m*_ and 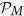 is illustrated in Fig. 3 whilst the time evolution of 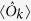 for a sample trajectory is shown in Fig. 2(a). These eigenspaces are composed of Fock states in momentum space that have the same number of atoms within each pair of *k* and *k* − *π/a* modes. The projectors 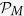 consist of many such eigenspaces leading to the case where we can only distinguish between the spaces that have different parities of 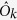.

### Experimental considerations

Before concluding this paper, it is worthwhile to consider the experimental difficulties in realising such an experiment. First, we note that there are two recent experiments that have successfully obtained an ultracold gas in an optical lattice coupled to a high-Q cavity^32^,^33^. The main major concern is photon detector inefficiency. It has been shown^31^ that as long as there is a sufficient number of photons detected such that the true instantaneous rate can be reliably estimated it is possible to use detectors with very low efficiencies. Another, possible issue is the sensitivity of the relative angle between the cavity and the probe beams. Generally, the most interesting arrangements, such as the two cases used in this paper, correspond to easily identifiable scattering features such as diffraction maxima and minima, and thus they should be easy to identify and tune. However, it is also possible to obtain identical jump operators with a homodyne detection scheme in which instead of angles, one has to tune the local oscillator phase which might potentially be easier to fine tune in an experiment^13^. Finally, one might also be concerned with possible dephasing due to scattering outside of the cavity. However, cavities used by experiments such as those in refs 17 and 33 have a Purcell factor of ~100 and probe-atom detunings in the MHz range. Thus, any scattering outside of the cavity can be safely neglected^17^.

## Discussion

In summary we have investigated measurement backaction resulting from coupling light to an ultracold gas’s phase-related observables. We demonstrated how this can be used to prepare the Hamiltonian eigenstates even if significant tunnelling is occuring as the measurement can be engineered to not compete with the system’s dynamics. Furthermore, we have shown that when the observable of the phase-related quantities does not commute with the Hamiltonian we still project to a specific subspace of the system that is neither an eigenspace of the Hamiltonian or the measurement operator. This is in contrast to quantum Zeno dynamics^49^,^50^,^51^,^52^,^53^ or dissipative state preparation^59^. We showed that this projection is essentially an extension of the measurement postulate to weak measurement on dynamical systems where the competition between the two processes is significant.

## Additional Information

**How to cite this article:** Kozlowski, W. *et al*. Quantum State Reduction by Matter-Phase-Related Measurements in Optical Lattices. *Sci. Rep.* **7**, 42597; doi: 10.1038/srep42597 (2017).

**Publisher's note:** Springer Nature remains neutral with regard to jurisdictional claims in published maps and institutional affiliations.

## Supplementary Material

Supplementary Information
