# Supplementary material for: Quantum State Reduction by Matter-Phase-Related Measurements in Optical Lattices
Source: Sci Rep. 2017 Feb 22;7:42597. doi: 10.1038/srep42597 (PMC5320499; doi:10.1038/srep42597)
Supplement: Supplementary Information [file srep42597-s1.pdf]

Supplementary Information: Quantum State Reduction by Matter-Phase-Related Measurements in Optical Lattices

Wojciech Kozlowski[1,*], **Santiago F. Caballero-Benitez**[1], **and Igor B. Mekhov**[1]

[1]Department of Physics, Clarendon Laboratory, University of Oxford, Parks Road, Oxford OX1 3PU, United Kingdom
[*]wojciech.kozlowski@physics.ox.ac.uk

ABSTRACT

In this Supplementary Information we provide additional details about the illumination patterns necessary to obtain $\hat{a} = C\hat{B}$, the derivation of the probability distribution for $\hat{B}_1$ and an explanation of how to identify the projection subspaces for $\hat{B}_2$.

S1 Suppressing the effective coupling to atomic density

In the main text we showed that $\hat{a} = C(\hat{D} + \hat{B})$, where

$$C = \frac{g_{\text{out}} g_{\text{in}} a_0}{\Delta_a (\Delta_p + i\kappa)}, \tag{S1}$$

$g_{\text{out,in}}$ are the atom-light coupling constants for the outgoing and incoming beams, Δ_a is the detuning between the incoming probe beam and the atomic resonance frequency, Δ_p is the detuning between the incoming probe beam and the outgoing cavity beam, a_0 is the amplitude of the coherent probe beam, and κ is the cavity decay rate. However, we are only interested in the case when $\hat{a} = C\hat{B}$. Therefore, we need to find the conditions under which this is true. For clarity we will consider a 1D lattice, but the results can be applied and generalised to higher dimensions. Central to engineering the $\hat{a}$ operator are the coefficients $J_{m,n}$ given by

$$J_{m,n} = \int w(\mathbf{r} - \mathbf{r}_m) u^*_{\text{out}}(\mathbf{r}) u_{\text{in}}(\mathbf{r}) w(\mathbf{r} - \mathbf{r}_n) \, \mathrm{d}\mathbf{r}, \tag{S2}$$

where $w(\mathbf{r})$ are the Wannier functions of the lowest band, $u_{\text{in,out}}(\mathbf{r})$ are the light mode functions of the incoming and outgoing beams, and $\mathbf{r}$ is the position vector. The operators $\hat{B}$ and $\hat{D}$ depend on the values of $J_{m,m+1}$ and $J_{m,m}$ respectively and are given by

$$\hat{D} = \sum_m^K J_{m,m} \hat{n}_m, \tag{S3}$$

$$\hat{B} = \sum_m^K J_{m,m+1} \left(b^\dagger_m b_{m+1} + b_m b^\dagger_{m+1} \right), \tag{S4}$$

where b_m annihilates an atom at site m, and $\hat{n}_m = b^\dagger_m b_m$ is the number operator at site m. These $J_{m,n}$ coefficients are determined by the convolution of the light mode product, $u^*_{\text{out}}(\mathbf{r}) u_{\text{in}}(\mathbf{r})$ with the relevant Wannier function overlap $w(\mathbf{r} - \mathbf{r}_m) w(\mathbf{r} - \mathbf{r}_n)$. For the $\hat{B}$ operator we calculate the convolution with the nearest neighbour overlap, $W_1(\mathbf{r}) \equiv w(\mathbf{r} - \mathbf{a}/2) w(\mathbf{r} + \mathbf{a}/2)$, where $\mathbf{a}$ is the site separation vector, and for the $\hat{D}$ operator we calculate the convolution with the square of the Wannier function at a single site, $W_0(\mathbf{r}) \equiv w^2(\mathbf{r})$. Therefore, in order to enhance the $\hat{B}$ term we need to maximise the overlap between the light modes and the nearest neighbour Wannier overlap, $W_1(\mathbf{r})$. This can be achieved by concentrating the light between the sites rather than at atom positions.

In order to calculate the $J_{m,n}$ coefficients it is necessary to perform numerical calculations using realistic Wannier functions. However, it is possible to gain some analytic insight into the behaviour of these values by looking at the Fourier transforms of the Wannier function overlaps, $\mathscr{F}[W_{0,1}](\mathbf{k})$. This is because the light mode product, $u^*_{\text{out}}(\mathbf{r}) u_{\text{in}}(\mathbf{r})$, can be in general decomposed into a sum of oscillating exponentials of the form $e^{i\mathbf{k}\cdot\mathbf{r}}$ making the integral in Eq. (S2) a sum of Fourier transforms of $W_{0,1}(\mathbf{r})$.

We consider a setup shown in Fig. S1 and take both the detected and probe beam to be standing waves, $u_{\text{in,out}}(\mathbf{r}) = \cos(\mathbf{k}_{\text{in,out}} \cdot \mathbf{r} + \varphi_{\text{in,out}})$, where $\mathbf{k}$ is the wavevector of the beam and φ is a constant phase shift. This gives the following expressions for the $\hat{D}$ and $\hat{B}$ operators

$$\hat{D} = \frac{1}{2}[\mathscr{F}[W_0](k_-)\sum_m \hat{n}_m \cos(k_- x_m + \varphi_-) + \mathscr{F}[W_0](k_+)\sum_m \hat{n}_m \cos(k_+ x_m + \varphi_+)], \tag{S5}$$

$$\hat{B} = \frac{1}{2}[\mathscr{F}[W_1](k_-)\sum_m \hat{p}_m \cos(k_- x_m + \frac{k_- a}{2} + \varphi_-) + \mathscr{F}[W_1](k_+)\sum_m \hat{p}_m \cos(k_+ x_m + \frac{k_+ a}{2} + \varphi_+)], \tag{S6}$$

where $x_m = ma$, $k_\pm = k_{\text{in},x} \pm k_{\text{out},x}$, $k_{(\text{in,out})x} = k_{\text{in,out}} \sin(\theta_{\text{in,out}})$, $\hat{p}_m = b_m^\dagger b_{m+1} + b_m b_{m+1}^\dagger$, and $\varphi_\pm = \varphi_{\text{in}} \pm \varphi_{\text{out}}$. The key result is that the $\hat{B}$ operator is phase shifted by $k_\pm d/2$ with respect to the $\hat{D}$ operator since it depends on the amplitude of light in between the lattice sites and not at the positions of the atoms, allowing to decouple them at specific angles.

Firstly, we will use this result to show how one can obtain the uniform pattern for which $\hat{B} = \hat{B}_1$, where

$$\hat{B}_1 = \sum_m^K J_1 \left(b_m^\dagger b_{m+1} + b_m b_{m+1}^\dagger \right), \tag{S7}$$

i.e. $J_{m,m+1} = J_1$ This can be achieved by crossing the light modes such that $\theta_{\text{in}} = -\theta_{\text{out}}$ and $k_{\text{in},x} = k_{\text{out},x} = \pi/a$ and choosing the light mode phases such that $\varphi_+ = \pi$. In order to make the $\hat{B}$ contribution to light scattering dominant we need to set $\hat{D} = 0$ which from Eq. (S5) we see is possible if $\varphi_- = \arccos[\mathscr{F}[W_0](2\pi/a)/\mathscr{F}[W_0](0)]/2$. This arrangement of light modes maximizes the interference signal, $\hat{B}$, by suppressing the density signal, $\hat{D}$, via interference compensating for the spreading of the Wannier functions and leads to the parameter value $J_1 = \mathscr{F}[W_1](2\pi/a)/2$. The light mode patterns are illustrated in the main text in Fig. 1(a).

Secondly, we show that we can have a spatially varying pattern for which $\hat{B} = \hat{B}_2$, where

$$\hat{B}_2 = \sum_m^K (-1)^m J_2 \left(b_m^\dagger b_{m+1} + b_m b_{m+1}^\dagger \right). \tag{S8}$$

We consider an arrangement where the beams are arranged such that $k_{\text{in},x} = 0$ and $k_{\text{out},x} = \pi/a$ which gives the following expressions for the density and interference terms

$$\begin{aligned} \hat{D} &= \mathscr{F}[W_0](\pi/a)\sum_m (-1)^m \hat{n}_m \cos(\varphi_{\text{in}})\cos(\varphi_{\text{out}}) \\ \hat{B} &= -\mathscr{F}[W_1](\pi/a)\sum_m (-1)^m \hat{p}_m \cos(\varphi_{\text{in}})\sin(\varphi_{\text{out}}). \end{aligned} \tag{S9}$$

It is clear that for $\varphi_{\text{out}} = \pm\pi/2$, $\hat{D} = 0$, which is intuitive as this places the lattice sites at the nodes of the mode $u_{\text{out}}(\mathbf{r})$ and yields the parameter value $J_2 = -\mathscr{F}[W_1](\pi/a)\cos(\varphi_{\text{in}})$. This is a diffraction minimum as the light amplitude is zero, $\langle \hat{B} \rangle = 0$, because contributions from alternating inter-site regions interfere destructively. However, the intensity $\langle \hat{a}^\dagger \hat{a} \rangle = |C|^2 \langle \hat{B}^2 \rangle$ is proportional to the variance of $\hat{B}$ and is non-zero. The light mode patterns are illustrated in the main text in Fig. 1(b).

S2 Finding the measurement projection subspaces

The main text defines the projectors $\mathscr{P}_M = \sum_{m \in M} P_m$, where P_m are the projectors onto the $\hat{a}$ eigenspaces, such that $\sum_M \mathscr{P}_M = \hat{1}$, $\mathscr{P}_M \mathscr{P}_N = \delta_{M,N} \mathscr{P}_M$, $[\mathscr{P}_M, \hat{H}_0] = 0$, and $[\mathscr{P}_M, \hat{a}] = 0$. To find $\mathscr{P}_M$ we need to identify the subspaces M which satisfy the following relation $\sum_{m \in M} P_m = \sum_{m \in M} |h_m\rangle\langle h_m|$, where $|h_m\rangle$ are the eigenstates of $\hat{H}_0$. This can be done iteratively by (i) selecting some P_m, (ii) identifying the $|h_m\rangle$ which overlap with this subspace, (iii) identifying any other P_m which also overlap with these $|h_m\rangle$ from step (ii). We repeat (ii)-(iii) for all the P_m found in (iii) until we have identified all the subspaces P_m linked in this way and they will form one of our $\mathscr{P}_M$ projectors. If $\mathscr{P}_M \neq 1$ then there will be other subspaces P_m which we have not included so far and thus we repeat this procedure on the unused projectors until we identify all $\mathscr{P}_M$. Computationally this can be straightforwardly solved with some basic algorithm that can compute the connected components of a graph.

The above procedure, whilst mathematically correct and always guarantees to generate the projectors $\mathscr{P}_M$, is very unintuitive and gives poor insight into the nature or physical meaning of $\mathscr{P}_M$. In order to get a better understanding of these subspaces we will use another result from the main text. We showed that for an operator $\hat{O}$ with eigenspace projectors R_m for which $[\hat{O}, \hat{H}_0] = 0$, and $[\hat{O}, \hat{a}] = 0$, then we can write the subspace projectors as $\mathscr{P}_M = \sum_{m \in M} R_m = \sum_{m \in M} P_m$.

We are interested in identifying these subspaces for the operator $\hat{B}_2$ given by

$$\hat{B}_2 = \sum_m^K (-1)^m J_2 \left(b_m^\dagger b_{m+1} + b_m b_{m+1}^\dagger \right)$$
$$= 2iJ_2 \sum_k c_k^\dagger c_{k-\pi/a} \sin(ka). \tag{S10}$$

We have identified that for $\hat{B}_2$, an operator $\hat{O}$ that commutes with both the measurement operator and the Hamiltonian is given by $\hat{O} = \sum_{\text{RBZ}} g_k \hat{O}_k$, where $\hat{O}_k = \hat{n}_k + \hat{n}_{k-\pi/a}$, for any arbitrary constants g_k. The subspaces R_m of this operator simply consist of momentum space Fock states that have the same number of atoms in each $(k, k-\pi/a)$ pair of momenta. However, it turns out that the $\mathscr{P}_M$ consist of multiple such subspaces complicating the picture.

Firstly, since $\hat{B}_2$ contains $\sin(ka)$ coefficients atoms in different k modes that have the same $\sin(ka)$ value are indistinguishable to the measurement and will lie in the same P_m eigenspaces. This will happen for the pairs $(k, \pi/a - k)$. Therefore, the R_m spaces that have the same $\hat{O}_k + \hat{O}_{\pi/a-k}$ eigenvalues must belong to the same $\mathscr{P}_M$.

Secondly, if we re-write these operators in terms of the β_k and $\tilde{\beta}_k$ modes we get

$$\hat{B}_2 = 2J_2 \sum_{\text{RBZ}} \sin(ka) \left(\beta_k^\dagger \beta_k - \tilde{\beta}_k^\dagger \tilde{\beta}_k \right), \tag{S11}$$

$$\hat{O} = \sum_{\text{RBZ}} g_k \left(\beta_k^\dagger \beta_k + \tilde{\beta}_k^\dagger \tilde{\beta}_k \right), \tag{S12}$$

and so it's not hard to see that $\hat{B}_{2,k} = (\beta_k^\dagger \beta_k - \tilde{\beta}_k^\dagger \tilde{\beta}_k)$ will have the same eigenvalues for different values of $\hat{O}_k = \beta_k^\dagger \beta_k + \tilde{\beta}_k^\dagger \tilde{\beta}_k$. Specifically, if a given subspace R_m corresponds to the eigenvalue O_k of $\hat{O}_k$ then the possible values of $B_{2,k}$ will be $\{-O_k, -O_k + 2, ..., O_k - 2, O_k\}$. Thus, we can see that all R_m with even values of O_k will share $B_{2,k}$ eigenvalues and thus they will overlap with the same P_m subspaces. The same is true for odd values of O_k. However, R_m with an even value of O_k will never have the same value of $B_{2,k}$ as a subspace with an odd value of O_k. Therefore, a single $\mathscr{P}_M$ will contain all R_m that have the same parities of O_k for all k, e.g. if it includes the R_m with $O_k = 6$, it will also include the R_m for which $O_k = 0, 2, 4, 6, ..., N$, where N is the total number of atoms.

Finally, the $k = \pi/a$ mode is special, because $\sin(\pi) = 0$ which means that $B_{2,k=\pi/a} = 0$ always. This in turn implies that all possible values of $O_{\pi/a}$ are degenerate to the measurement. Therefore, we exclude this mode when matching the parities of the other modes.

To illustrate the above let us consider a specific example. Let us consider two atoms, $N = 2$, on eight sites $M = 8$. This configuration has eight momentum modes $ka = \{-\frac{3\pi}{4}, -\frac{\pi}{2}, -\frac{\pi}{4}, 0, \frac{\pi}{4}, \frac{\pi}{2}, \frac{3\pi}{4}, \pi\}$ and so the RBZ has only four modes RBZ $:= \{\frac{\pi}{4}, \frac{\pi}{2}, \frac{3\pi}{4}, \pi\}$. There are 10 different ways of splitting two atoms into these four modes and thus we have 10 different $R_m = \{O_{\pi/4a}, O_{\pi/2a}, O_{3\pi/4a}, O_{\pi/a}\}$ eigenspaces of $\hat{O}$ and they are shown in Table S1. In the third column we have also listed the eigenvalues of the $\hat{B}_2$ eigenstates that lie within the given R_m.

We note that $ka = \pi/4$ will be degenerate with $ka = 3\pi/4$ since $\sin(ka)$ is the same for both. Therefore, we already know that we can combine (R_0, R_2, R_7), (R_1, R_5), and (R_3, R_8), because those combinations have the same $O_{\pi/4a} + O_{3\pi/4a}$ values. This is very clear in the table as these subspaces span exactly the same values of B_2.

Now we have to match the parities. Subspaces that have the same parity combination for the pair $(O_{\pi/4a} + O_{3\pi/4a}, O_{\pi/2a})$ will be degenerate in $\mathscr{P}_M$. Note that we excluded $O_{\pi/a}$, because as we discussed earlier they are all degenerate due to $\sin(\pi) = 0$. Therefore, the (even,even) subspace will include $(R_0, R_2, R_4, R_7, R_9)$, the (odd,even) will contain (R_3, R_8), the (even, odd) will contain (R_6) only, and the (odd, odd) contains (R_1, R_5). These overlaps should be evident from the table as we can see that these combinations combine all R_m that contain any eigenstates of $\hat{B}_2$ with the same eigenvalues.

Therefore, we have end up with four distinct $\mathscr{P}_M$ subspaces

$$\mathscr{P}_{\text{even,even}} = R_0 + R_2 + R_4 + R_7 + R_9$$
$$\mathscr{P}_{\text{odd,even}} = R_3 + R_8$$
$$\mathscr{P}_{\text{even,odd}} = R_6$$
$$\mathscr{P}_{\text{odd,odd}} = R_1 + R_5.$$

At this point it should be clear that these projectors satisfy all our requirement. The conditions $\sum_M \mathscr{P}_M = 1$ and $\mathscr{P}_M \mathscr{P}_N = \delta_{M,N} \mathscr{P}_M$ should be evident from the form above. The commutator requirements are also easily satisfied since the subspaces R_m are of an operator that commutes with both the Hamiltonian and the measurement operator. And finally, one can also verify using the table that all of these projectors are built from complete subspaces of $\hat{B}_2$ (i.e. each subspace P_m belongs to only one $\mathscr{P}_M$) and thus $\mathscr{P}_M = \sum_{m \in M} P_m$.

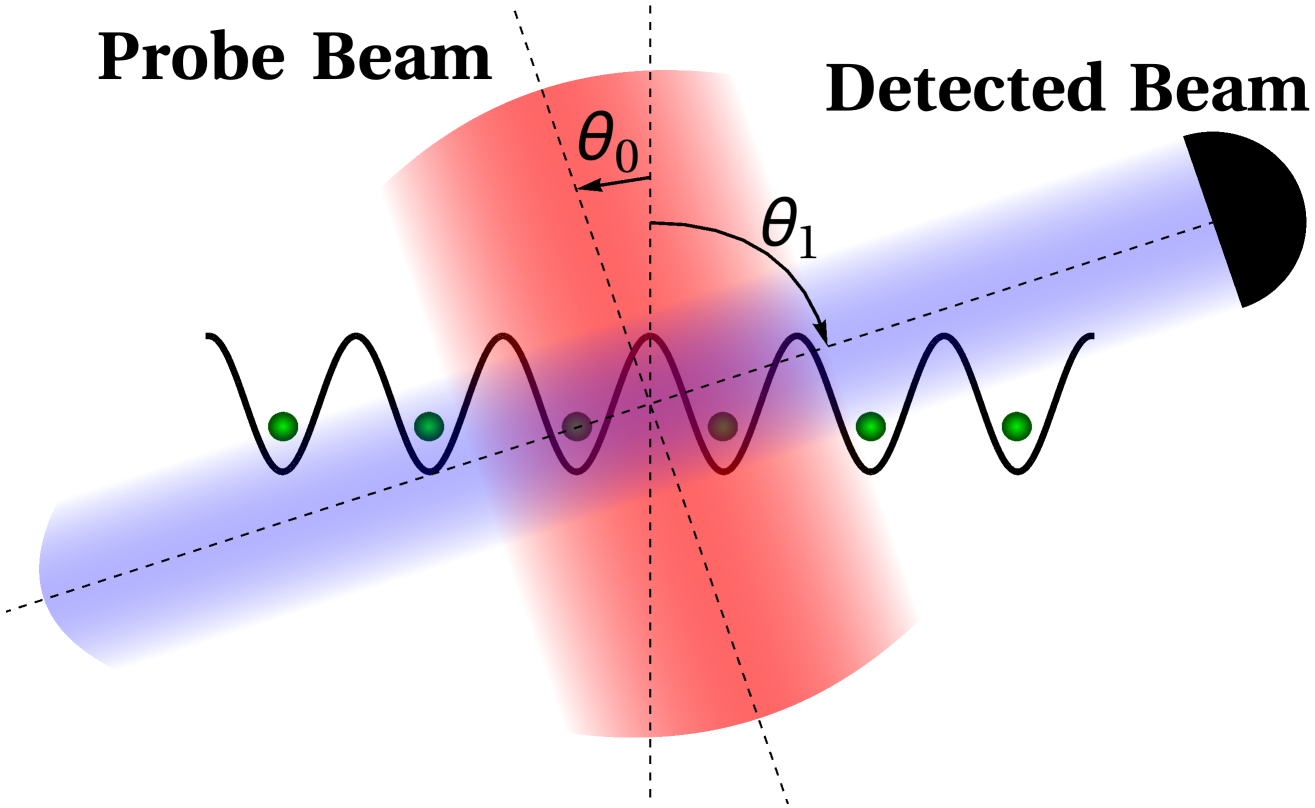

Figure S1. Setup. Atoms in an optical lattice are illuminated by a probe beam. The light scatters in free space or into a cavity and is measured by a detector.

m	R_m	Possible values of $B_{\min}$
0	$\{2,0,0,0\}$	$-\sqrt{2}, 0, \sqrt{2}$
1	$\{1,1,0,0\}$	$-\frac{1+\sqrt{2}}{\sqrt{2}}, -\frac{1-\sqrt{2}}{\sqrt{2}}, \frac{1-\sqrt{2}}{\sqrt{2}}, \frac{1+\sqrt{2}}{\sqrt{2}}$
2	$\{1,0,1,0\}$	$-\sqrt{2}, 0, \sqrt{2}$
3	$\{1,0,0,1\}$	$-\frac{1}{\sqrt{2}}, \frac{1}{\sqrt{2}}$
4	$\{0,2,0,0\}$	$-2, 0, 2$
5	$\{0,1,1,0\}$	$-\frac{1+\sqrt{2}}{\sqrt{2}}, -\frac{1-\sqrt{2}}{\sqrt{2}}, \frac{1-\sqrt{2}}{\sqrt{2}}, \frac{1+\sqrt{2}}{\sqrt{2}}$
6	$\{0,1,0,1\}$	$-1, 1$
7	$\{0,0,2,0\}$	$-\sqrt{2}, 0, \sqrt{2}$
8	$\{0,0,1,1\}$	$-\frac{1}{\sqrt{2}}, \frac{1}{\sqrt{2}}$
9	$\{0,0,0,2\}$	0

Table S1. A list of all R_m eigenspaces for $N = 2$ atoms at $M = 8$ sites. The third column displays the eigenvalues of all the eigenstates of $\hat{B}_2$ that lie in the given R_m.
